# Eye-tracking-based tablet application for enhanced cognitive assessment: a clinical trial for software as a medical device (SaMD)

**DOI:** 10.1007/s11357-025-01941-x

**Published:** 2025-10-20

**Authors:** Yoichi Takami, Tetsuro Miki, Yasushi Takeya, Hiromi Rakugi, Ryuichi Morishita, Shuko Takeda

**Affiliations:** 1 Department of Geriatric and General Medicine, Graduate School of Medicine, Osaka University, Suita, Osaka 565-0871 Japan; 2 Hanwa Daiichi Senboku Hospital, Sakai, Osaka 590-0106 Japan; 3 Department of Clinical Nursing Division of Health Sciences Graduate School of Medicine, Osaka University, Suita, Osaka 565-0871 Japan; 4 Department of Clinical Gene Therapy, Graduate School of Medicine, Osaka University, 2-2 Yamada-Oka, Suita, Osaka 565-0871 Japan

**Keywords:** Clinical trial, Cognitive assessment, Dementia, Eye-tracking, Medical device

## Abstract

Efficient screening tools for cognitive decline and dementia are in high demand. We previously reported on a novel cognitive assessment tool using eye-tracking technology and its utility in dementia screening. In this study, we developed a tablet version of the eye-tracking-based cognitive assessment (ETCA) app and conducted a clinical trial to verify its clinical utility as software as a medical device (SaMD). Participants with and without dementia were enrolled between December 1, 2020, and July 31, 2021. They used the ETCA app and completed the most widely applied screening test, the Mini-Mental State Examination (MMSE), on the same day. The primary outcome was the correlation between the ETCA and MMSE scores. The key secondary outcomes were the administration time for the ETCA, questionnaire-based assessments of test-related burdens, and correlations of the subscores. Performance for detecting dementia was evaluated exploratorily. Analyses of the full analysis set population revealed a strong positive correlation in total scores between the tests (r = 0.831; 95% CI, 0.743–0.891; *p* < 0.0001). The median ETCA administration time was 254.0 s, and more than 70% of participants reported experiencing less or a similar test-related burden with the ECTA app, compared to the MMSE. Significant correlations were observed for all subscores. The ETCA achieved an area under the curve of 0.864 in detecting participants with dementia. These results verified the clinical utility of the tablet-based ETCA app as a rapid cognitive assessment tool. Based on the results, the ETCA app has obtained regulatory approval as SaMD.

**Supplementary Information:**

The online version contains supplementary material available at https://doi.org/10.1007/s11357-025-01941-x.

## Introduction

Dementia is becoming an increasingly serious social issue worldwide, and recent literature suggests that early diagnosis and intervention can preventits progression or onset [1]. The development of a disease-modifying treatment for Alzheimer’s disease, which targets the early stage of the disease, adds weight to early or timely diagnostic efforts [2, 3]. However, most people with dementia remain undiagnosed, with only 20%–50% of cases recognized [4]. The primary reason for this low diagnosis rate is the lack of an efficient tool to help general practitioners screen for cognitive decline and dementia.

The first step toward diagnosing dementia involves a cognitive function assessment that employs interview-based neuropsychological tests. The Mini-Mental State Examination (MMSE) is a method commonly used by specialists and general practitioners in medical science because of its long history, high validity, and reliability in detecting cognitive decline, as well as its ability to assess the major cognitive domains [5, 6]. The MMSE and other neuropsychological tests enable the quantitative assessment of the core symptoms of dementia and have a well-established clinical utility [7].

Despite their reliability for dementia screening, cognitive tests like the MMSE have certain limitations. For instance, the MMSE takes 10–20 min to complete and may take longer for elderly patients, making it unsuitable for routine clinical screening [8]. Furthermore, subjects may experience fatigue and stress during the assessment, and the administrator’s proficiency level can significantly influence the results. This highlights the need for more efficient and convenient assessment tools for addressing the worldwide dementia epidemic.

Digital technologies have transformed traditional neuropsychological tests into more efficient methods for dementia screening [9, 10]. We previously developed an innovative digital cognitive assessment tool using eye-tracking technology and reported on its utility in dementia screening [11, 12]. The eye-tracking-based cognitive assessment (ETCA) [11–13] uses eye-tracking technology for gaze detection and task movies designed to evaluate the major cognitive domains. It records the subject’s gaze during a task and quantifies their cognitive function based on the recorded gaze data. We demonstrated good correlations between ETCA scores and those of the MMSE and other neuropsychological tests, indicating the effectiveness of the ETCA for detecting cognitive deficits [11, 12]. The ETCA has several advantages over traditional neuropsychological tests: 1) it is a time-efficient assessment method, compared to interview-based tests; 2) it is less stressful for the participant, as they only need to view task movies for assessment; 3) it does not require highly trained medical staff to administer the test; and 4) the rater’s skill or knowledge does not influence the results, as the score is automatically calculated based on gaze data.

Aiming to enhance the ETCA’s cost-effectiveness and scalability, we developed a tablet-based version of the ETCA app that uses a tablet’s built-in camera for eye tracking. The app presents task movies (3 min in length) to assess the major cognitive domains, such as orientation, attention, memory, language, and visuospatial function, on the tablet’s screen. The app calculates the cognitive score based on the gaze information and provides a score upon completion of the task movies. In the present study, we conducted a multicenter clinical trial to verify the tablet-based ETCA app’s clinical utility and to obtain regulatory approval for it as software as a medical device (SaMD). The trial examined the ETCA’s correlation with MMSE scores, performance in detecting dementia, administration time, and test-related burdens. This clinical trial is the first to demonstrate the performance and clinical utility of the tablet-based ETCA app.

## Methods

### Trial design and participants

This single-arm, open-label, uncontrolled, multicenter clinical trial was conducted at two medical facilities in Japan between December 1, 2020, and July 31, 2021. Its objective was to verify the correlation between the scores obtained using the tablet-based ETCA app and the MMSE. Trial participants were patients clinically diagnosed with dementia, subjects with suspected MCI, and cognitively healthy controls. The Osaka University Hospital’s Institutional Review Board approved the study protocol, and the Ethics Committee of Osaka University provided ethical approval. The study followed all relevant guidelines and regulations, including the principles of the Declaration of Helsinki. Written informed consent documents were obtained from all participants. Details on registration and sample-size calculation are provided in the supplementary information.

### Trial registration

Japan Registry of Clinical Trials: jRCT2052210004.

### Eligibility criteria

The main inclusion criteria were: 1) subjects aged 20 years or older, 2) subjects who had been clinically diagnosed as having dementia, were suspected of having MCI, or who were cognitively normal. Subjects with a history of active neurological disorders or psychological disease were excluded. A diagnosis of dementia was made by the principal investigators, who are experts in geriatric medicine and dementia, based on the criteria defined in the *DSM-IV *[14].

The main exclusion criteria were: 1) Participants for whom gaze detection was impossible because of severe visual impairments (e.g., low visual acuity due to conditions such as strabismus or cataracts, or visual field defects such as glaucoma or diabetes). Participants who wear glasses with an extremely small or thick frame, high-index lenses, or bifocal or multifocal lenses (convex lenses) were required to take the ETCA without glasses, as these types of lenses can make eye-tracking difficult and influence scoring. 2) Participants with any neurological disorders with dyskinesia or hearing loss, rendering administration of the MMSE difficult. 3) Participants who had completed the ETCA or MMSE within 3 months of enrollment in the study. 4) Participants who were judged by the investigators as ineligible for participation in the trial for other reasons (e.g., those for whom gaze data could not be properly measured, due to eye nystagmus or trauma or those with difficulty maintaining posture). Participants for whom criteria 1), 2), and/or 4) apply would be difficult to assess using the ETCA and MMSE, for mechanical reasons; the ETCA and MMSE scores of participants affected by criterion 3) might be influenced by previous testing.

No restrictions were imposed on medicine or therapy use, including antidementia medications (e.g., cholinesterase inhibitors and memantine). However, participants were not allowed to take any medications during the assessments (from the initiation of the MMSE through completion of the ETCA).

### Assessment of the ETCA application

The ETCA app consists of three elemental functions: 1) to display programmed task movies designed for cognitive assessment on a screen, 2) to continuously detect and record the subject’s gaze information (time-course plane coordinates) while viewing the task movies, and 3) to calculate and output the scores based on the subject’s viewing time (% of total time) in the region of interest (ROI) set on the correct image. The scores from each task were averaged and used as the total score. After completing the task movie, the total score and subscores were presented on the screen.

The programmed task movies consisted of an instruction/question with four distinct images as options to choose from, including one correct answer and other distractors (Supplementary Fig. 1). The program instructs the subject to find and focus on the correct answer. Based on the subject’s viewing time (% of total time) for correct answers on the task movies, the ETCA app scores each cognitive domain (i.e., orientation, attention/judgment, memory, language, and visuospatial function) and outputs the total score of the assessment.

The ETCA app’s eye-tracking system consists of an algorithm that uses facial recognition based on images taken from the built-in camera of a general tablet device. The ETCA app operates on an iPad Pro running the iPadOS and has an eye-tracking resolution of 50 Hz/s or greater. The optimal distance between the subject and the screen was 51 cm (a facial position guide was included to assist the subject in assuming the appropriate distance from and position relative to the screen).

The detailed information for data collection and processing, preparation for the assessment, calibration for eye tracking, and the task movies are presented in the supplementary information.

### Questionnaire-based assessments for participants and administrators

Details on the questionnaire-based assessments of participants’ fatigue and mental stress during the cognitive tests (conducted after completing the ETCA) are presented in the supplementary information.

### Safety evaluation

This study did not include any check/investigation items for safety evaluations, apart from adverse events or malfunctions. However, we did record information concerning those who failed to pass the calibration check 12 times (3 cycles × 4 sets), those who discontinued the trial, and those who did not complete the cognitive assessment for any reason. The investigators collected and recorded information about adverse events and malfunctions of the ETCA app related to each participant, as needed.

### MMSE subscores

We grouped the individual items on the MMSE into five cognitive domains based on previous research [15–18]. Each MMSE subscore was defined as follows. The mean scores of orientation to time (0–5 points) and place (0–5 points) were used as the orientation subscore; the mean scores of calculation (serial sevens, 0–5 points) and three-word registration (0–3 points) were used as the attention subscore; the score for delayed recall (0–3 points) was used as the memory subscore; the mean scores of naming (0–2 points), repetition (0–1 points), comprehension of a three-step command (0–3 points), sentence writing (0–1 points), and reading (0–1 points) were used as the language subscore; and the score of copying intersecting pentagons was used as the visuospatial function subscore.

### Statistical analyses

Regarding the primary outcome (the correlation in total scores between the ETCA and MMSE), the null hypothesis, H0, assumed that the lower limit of the two-sided 95% CI of the Spearman’s correlation coefficient was smaller than 0.55 in the cognitive scores between the MMSE and the ETCA app. The rationale for the criteria and sample-size calculation for the primary outcome are described in detail in the supplementary information (Methods, Sample-size calculation). Regarding the correlations in subscores between the ETCA and MMSE as the secondary outcomes, we evaluated p-values based on the results from the Spearman’s test. Differences in the participants’ ages, educational histories, ETCA app total scores, and the administration time among the three MMSE subgroups were analyzed using the Kruskal–Wallis test, followed by the post-hoc Steel–Dwass test for multiple comparisons. A two-sided chi-squared test was employed to test frequency differences among the groups. The performance of the ETCA app for detecting cognitive impairment or dementia was determined via ROC analysis. The AUC was used as an index of performance. The optimal cutoff point was determined using the maximal Youden index.　Statistical analyses were conducted using SAS software, version 9.4 (SAS Institute Inc.) and reviewed by external statistical experts (CMIC Holdings Co., Ltd.) for objectivity and reliability. A *p*-value < 0.05 was considered statistically significant.

## Results

### Participants

**Fig. 1:**
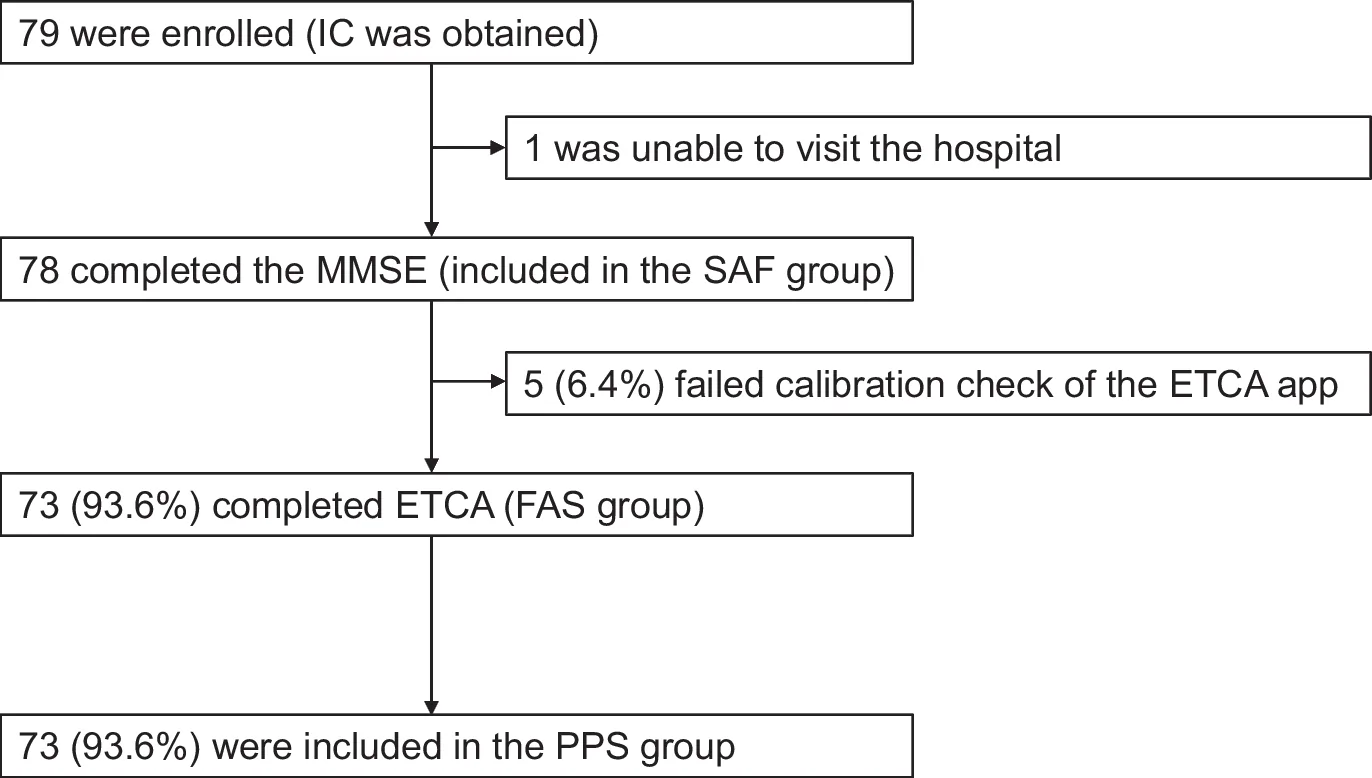
Study design. IC, informed consent; SAF, safety analysis set; FAS, full analysis set; PPS, per protocol set

This study recruited 79 participants from two medical institutions in Japan between December 1, 2020, and July 31, 2021 (Fig. 1). One participant was unable to visit the assigned site, due to COVID-19 restrictions at the facility, but 78 participants completed the MMSE. These 78 participants were included in the safety analysis set (SAF). Of the 78 participants, 73 (93.6%) completed the ETCA app assessment and were included in the full analysis set (FAS), while five failed to pass the ETCA app calibration check and were excluded from the primary outcome analysis. No protocol deviations were observed for the 73 participants in the FAS group; therefore, all were included in the per protocol set (PPS).

The participants’ characteristics are presented in Table 1. The FAS group consisted of 73 participants (43 women [58.9%], 30 men [41.1%]; mean age 77.4 (SD 7.7), range: 48–89). The mean ages of the MMSE subgroups were 78.5 (SD 7.7) for those with MMSE scores ≤ 19 (n = 28, 38.4%), 79.7 (SD 4.0) for those with MMSE scores of 20–26 (n = 21, 28.8%), and 74.0 (SD 10.8) for those with MMSE scores of ≥ 27 (n = 24, 32.9%). No significant differences in age were noted among the subgroups (*p* = 0.22). Thirty-nine (53.4%) participants in the FAS group were given a clinical diagnosis of dementia, while 34 (46.6%) did not have dementia. More details on the participants’ demographics are presented in Supplementary Table 1.

**Table 1:** Demographics and baseline characteristics (FAS population)

		MMSE ≤ 19	MMSE 20–26	27 ≤ MMSE	All	*p*-value
	Statistic	(*N* = 28)	(*N* = 21)	(*N* = 24)	(*N* = 73)	
Age, years	Mean (SD)	78.5 (5.7)	79.7 (4.0)	74.0 (10.8)	77.4 (7.7)	*p* = 0.22
Sex						
Male	n (%)	7 (25.0%)	8 (38.1%)	15 (62.5%)	30 (41.1%)	*p* < 0.05
Female	n (%)	21 (75.0%)	13 (61.9%)	9 (37.5%)	43 (58.9%)	
Educational history, years	Mean (SD)	11.9 (2.3)	11.8 (2.1)	14.0 (3.1)	12.6 (2.7)	*p* < 0.05
Status of dementia						
Dementia	n (%)	25 (89.3%)	14 (66.7%)	0 (0.0%)	39 (53.4%)	
Nondementia	n (%)	3 (10.7%)	7 (33.3%)	24 (100.0%)	34 (46.6%)	

### Cognitive assessment using the tablet-based ETCA app

**Fig. 2:**
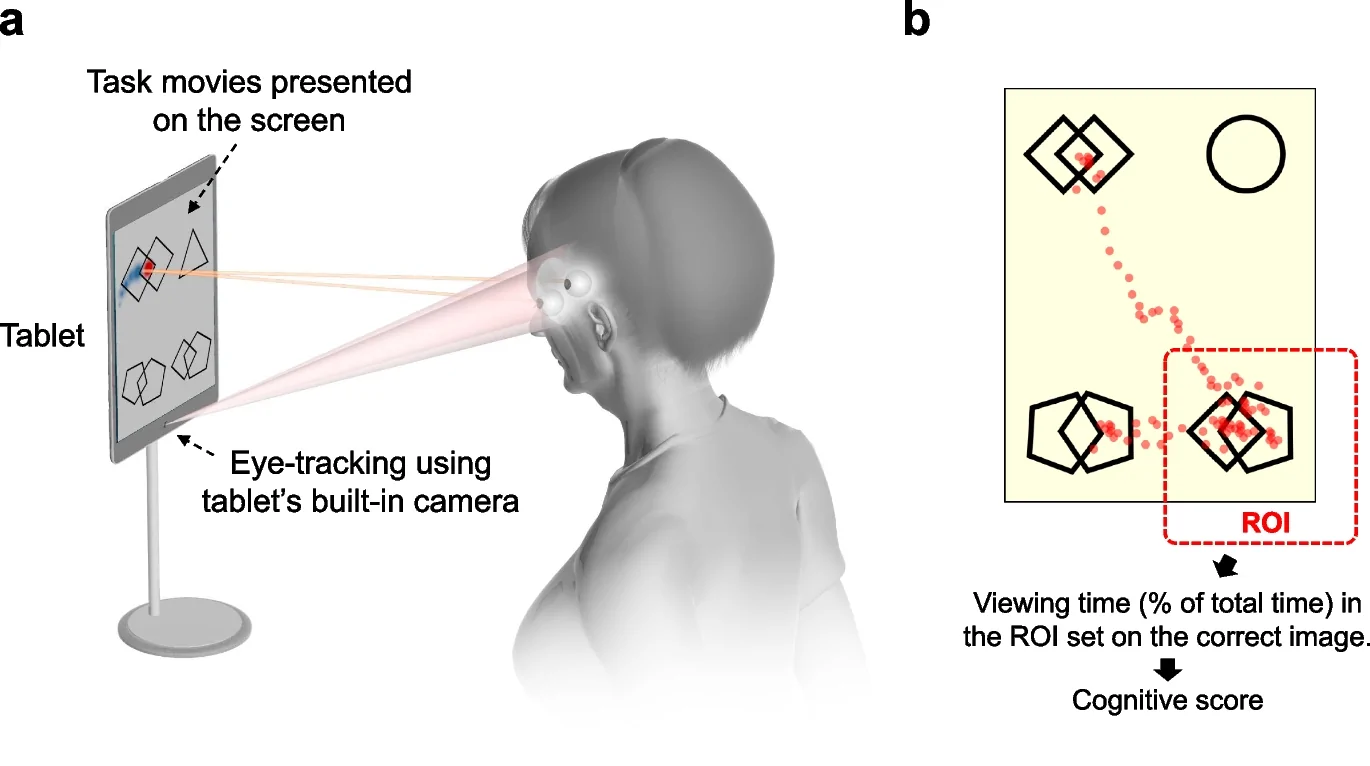
Overview of the ETCA app. **a** Schematic of the ETCA app. **b** Representative gaze plot data on the visuospatial function task. ROI, region of interest

Figure 2 presents an overview of the tablet-based ETCA app. The task movies (see Supplementary Fig. 1 for details) were presented on the screen and the participants’ gaze information was recorded using the tablet’s built-in camera (Fig. 2a). The cognitive scores were determined based on the participant’s viewing time (% of total time) in the region of interest set on the correct image (Fig. 2b). Details on the assessment by the ETCA app are presented in the supplementary information.

### Primary outcome

#### Correlation in total scores between the MMSE and the ETCA app

**Fig. 3:**
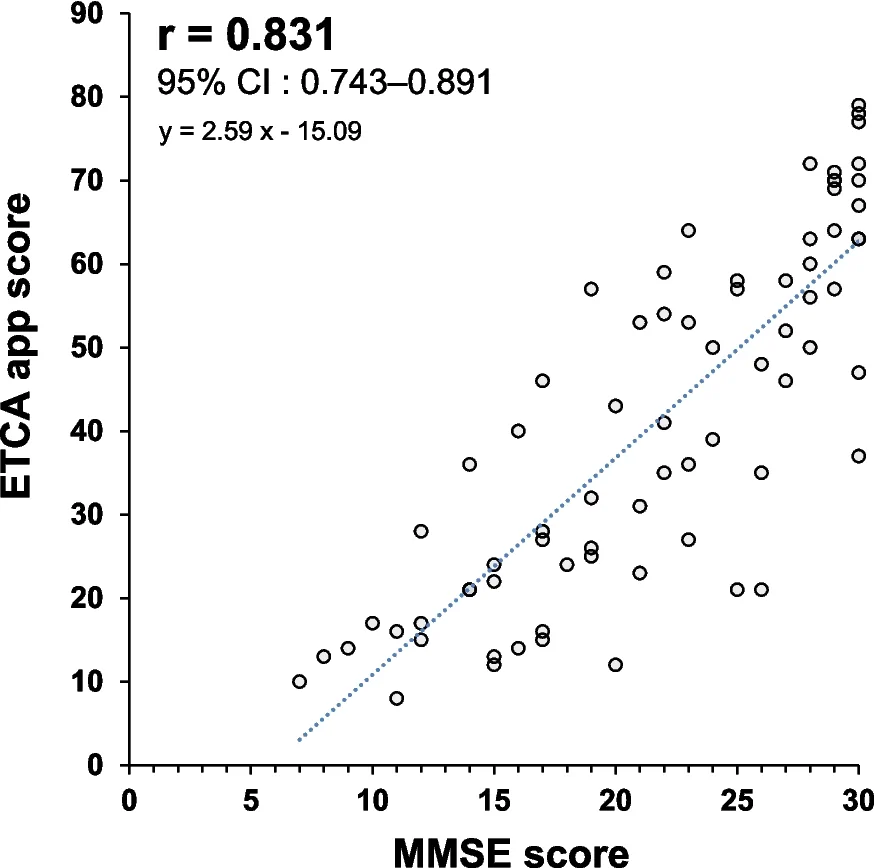
Correlation in the total scores between the MMSE and the ETCA app. Spearman’s rank correlation coefficient and two-sided 95% confidence interval (CI) are shown (*n* = 73, FAS/PPS population)

Figure 3 presents the correlation between the total scores for the ETCA app and MMSE. Analyses of the FAS/PPS population revealed a correlation coefficient of 0.831 (95% CI 0.743–0.891, *p* < 0.0001, Spearman) which met the pre-specified threshold of the 95% CI for the primary outcome, thereby demonstrating a strong positive correlation between the tests. The ETCA scores in each MMSE subgroup demonstrated significant differences among the subgroups (*p* < 0.001), reflecting the cognitive status of each subgroup with minimal overlap (Supplementary Fig. 2).

### Secondary outcomes

#### Correlations in subscores between the MMSE and the ETCA app

**Fig. 4:**
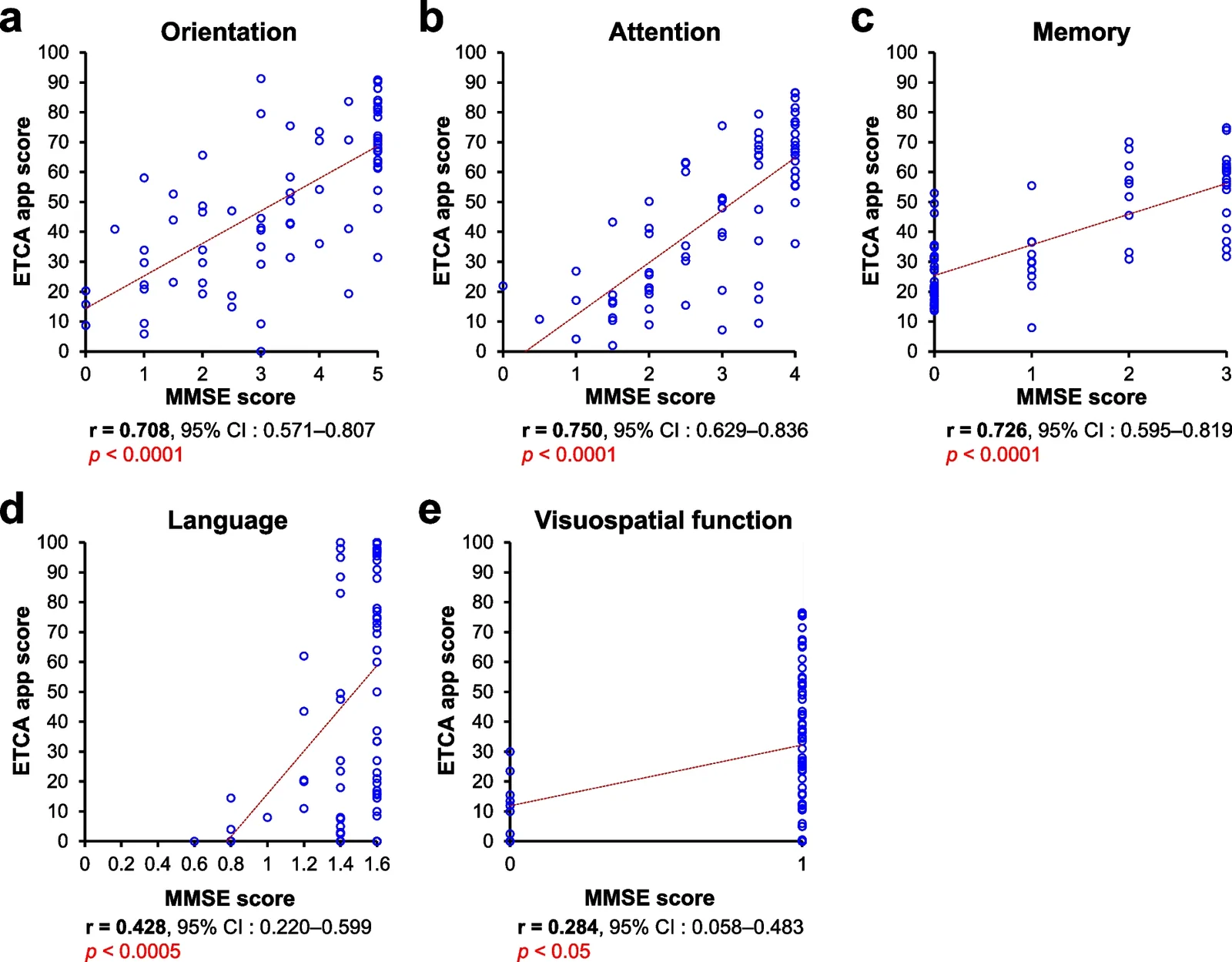
Scatter plot analyses for correlation in the subscores between the MMSE and the ETCA app. Scatter plots with Spearman’s rank correlation coefficient and its two-sided 95% confidence intervals (CI) for (**a**) orientation, **b** attention, **c** memory, **d** language, and (**e**) visuospatial function (e) are shown (n = 73, FAS/PPS population). *P*-values from the Spearman’s test for each correlation are also shown in red. Definitions of each MMSE subscore were described in the methods

Similar to the MMSE, the ETCA app provides subscores for each cognitive domain. Significant positive correlations were observed between the MMSE and ETCA app subscores (Table 2 and Fig. 4). Particularly high correlations were observed for orientation (r = 0.708 [95% CI 0.571–0.807]), attention (r = 0.750 [95% CI 0.629–0.836]), and memory (r = 0.726 [95% CI 0.595–0.819]). Relatively weak, yet still significant, correlations were observed for language (r = 0.428 [95% CI 0.220–0.599]) and visuospatial function (r = 0.284 [95% CI 0.058–0.483]), as only a single task is assigned for language and visuospatial function in the ETCA app and for visuospatial function in the MMSE. Notably, scatterplot analyses for these domains revealed that participants with lower scores on the MMSE in language and visuospatial function also had low scores on the ETCA app, reflecting impairment in those functions (Fig. 4).

**Table 2:** Correlation in the subscores between the MMSE and the ETCA app

	ETCA app				
MMSE	Orientation	Attention	Memory	Language	Visuospatial function
Orientation	0.708***	-	-	-	-
	(0.571–0.807)	-	-	-	-
Attention	-	0.750***	-	-	-
	-	(0.629–0.836)	-	-	-
Memory	-	-	0.726***	-	-
	-	-	(0.595–0.819)	-	-
Language	-	-	-	0.428**	-
	-	-	-	(0.220–0.599)	-
Visuospatial function	-	-	-	-	0.284*
	-	-	-	-	(0.058–0.483)

Spearman’s rank correlation coefficient and two-sided 95% CI are shown (*n* = 73, FAS/PPS population). *p < 0.05, **p < 0.0005, ***p < 0.0001

### Administration time and test-related burdens

The median administration time for the ETCA app was 254.0 s (Table 3). The mean administration times for the MMSE subgroups were 259.0 s for those with MMSE scores ≤ 19, 253.0 s for those scoring 20–26, and 254.0 s for those with scores 27 ≤. No significant differences were detected among the subgroups (*p* = 0.26).

**Table 3:** Administration time and questionnaire-based assessments of test-related burdens of the ETCA app

		MMSE ≤ 19	MMSE 20–26	27 ≤ MMSE	All	*p-*value
	Statistic	(*N* = 28)	(*N* = 21)	(*N* = 24)	(*N* = 73)	
Administration time						
Administration time of the ETCA app, seconds (SD)	n	28	21	24	73	
	Median	259.0	253.0	254.0	254.0	*p* = 0.26
	Mean	334.0 (161.7)	278.2 (81.2)	288.6 (120.0)	303.0 (129.9)	
Questionnaire-based assessments of fatigue and mental stress experienced by the participants						
Feelings of fatigue during the ETCA						
Felt less fatigued than during the MMSE	n (%)	3 (10.7%)	6 (28.6%)	9 (37.5%)	18 (24.7%)	
Felt the same as during the MMSE	n (%)	17 (60.7%)	9 (42.9%)	12 (50.0%)	38 (52.1%)	
Felt more fatigued than during the MMSE	n (%)	8 (28.6%)	6 (28.6%)	3 (12.5%)	17 (23.3%)	
Mental stress during the ETCA						
Felt less mental stress compared with the MMSE	n (%)	5 (17.9%)	5 (23.8%)	8 (33.3%)	18 (24.7%)	
Felt the same mental stress as with the MMSE	n (%)	13 (46.4%)	11 (52.4%)	10 (41.7%)	34 (46.6%)	
Felt more mental stress than with the MMSE	n (%)	10 (35.7%)	5 (23.8%)	6 (25.0%)	21 (28.8%)	

The results of the questionnaire-based assessments of the fatigue and mental stress experienced by the participants are presented in Table 3. In the FAS population, 76.8% (56/73) reported that their level of fatigue when using the ETCA app was the same as (52.1%) or less than (24.7%) when completing the MMSE. Additionally, 71.3% (52/73) reported that their mental stress when using the ETCA app was the same as (46.6%) or less than (24.7%) when completing the MMSE.

The questionnaire-based assessments for administrators regarding the ease of use of the ETCA app are presented in Supplementary Table 2. In 71 out of 73 individual assessments (97.2%), the administrators reported that the administration of the ETCA app was “easier” (84.2%) or “a little bit easier” (12.3%) than administering the MMSE. In a detailed reflective questionnaire after the trial’s completion, all administrators (4/4, 100%) reported that the ETCA app was both easier to use than the MMSE and could be used as an alternative to it (Supplementary Table 3).

### Performance of the ETCA app for detecting dementia

**Fig. 5:**
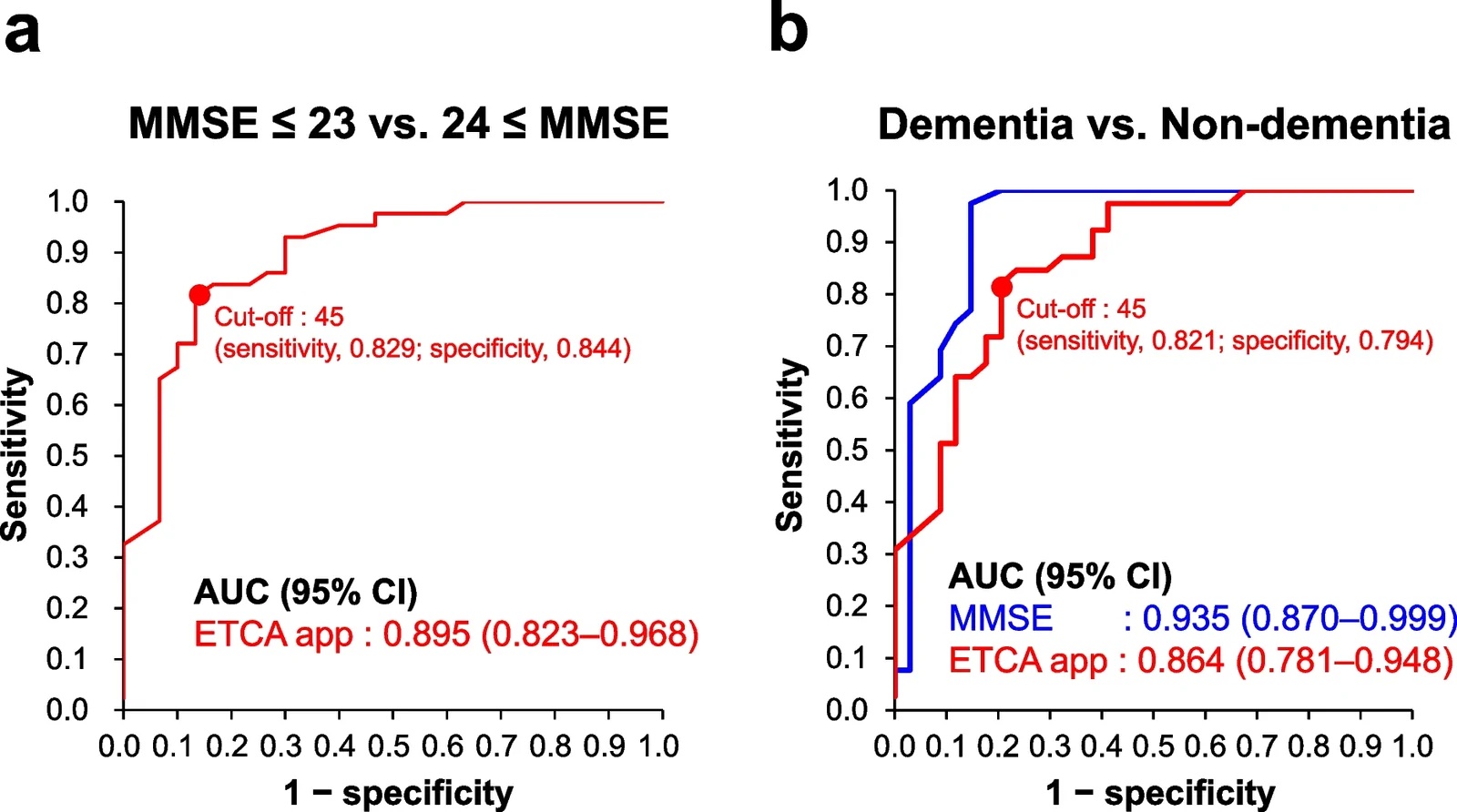
Diagnostic performance of the ETCA app for cognitive decline and dementia. **a** Receiver operating characteristic (ROC) curve for the ETCA app score to discriminate subjects with cognitive decline (MMSE ≤ 23) from those with MMSE ≥ 24. **b** ROC curves for ETCA app (red) and MMSE (blue) scores to discriminate subjects clinically diagnosed with dementia. Optimal cutoff values and their sensitivity and specificity are shown on the ROC curves. AUC, area under the curve; CI, confidence interval

The performance of the ETCA app for detecting cognitive impairment and dementia was assessed with a receiver operating characteristic (ROC) analysis (Fig. 5). The traditional cutoff value of ≤ 23/30 used for the MMSE to detect dementia reportedly yields sensitivity and specificity values greater than 0.8 [19, 20]. In the FAS population, 41 (56.2%) of the 73 participants had an MMSE score ≤ 23 and were thus regarded as having cognitive impairment. The ETCA app achieved excellent discrimination performance for detecting participants with cognitive impairment, with an area under the curve (AUC) of 0.895 (95% CI 0.823–0.968); the optimal cutoff value of 45/100 yielded a sensitivity of 0.829 and a specificity of 0.844 (Fig. 5a). The performances for detecting dementia using the ETCA app and MMSE are presented in Fig. 5b; the ETCA app achieved an AUC of 0.864 (95% CI 0.781–0.948), while the MMSE achieved an AUC of 0.935 (95% CI 0.870–0.999). The optimal cutoff value of 45/100 yielded a sensitivity of 0.821 and a specificity of 0.794 (positive predictive value (PPV), 0.821; negative predictive value (NPV), 0.794) for the ECTA app’s discrimination between dementia and non-dementia participants. The MMSE’s cutoff value of 23 (or less) yielded a sensitivity of 0.974 and a specificity of 0.853 (PPV, 0.884; NPV, 0.967) for detecting dementia.

### Safety evaluation

Mild neck pain was reported during the ETCA app assessment in one (1.3%) of the 78 SAF participants. We classified this as an adverse event, as a causal relationship could not be excluded (Supplementary Table 4). That participant spontaneously recovered within the day without any treatment. No serious adverse events were reported during the study. Malfunctions of the ETCA app occurred for 2 (2.6%) of the 78 participants (Supplementary Table 5), with one reporting transient flickering of the monitor during the assessment and the other reporting a monitor freeze during calibration. The ETCA assessments were completed in both cases, and scores were obtained without defects in the gaze plots needed to calculate the scores.

## Discussion

Our previous study, which used a research-based eye-tracking device, demonstrated a significant correlation between the MMSE and ETCA in a laboratory-based environment [11]. For this study, we developed a tablet-based ETCA app to enhance the tool’s cost-effectiveness and scalability and conducted a clinical trial to generate robust clinical evidence for its regulatory approval as SaMD. The results verified the high correlation in scores between the newly developed tablet-based ETCA app and the most widely applied assessment tool, the MMSE (Fig. 3) [21]. Most participants completed the ETCA app assessment within 4–5 min and reported a reduced test-related burden, compared to the MMSE (Table 3).

Eye-tracking technology has been used extensively in neuroscience and dementia research [5–10]. In such studies, a subject’s gaze is analyzed to calculate gaze-related metrics, including viewing time for specific objects or basic oculomotor features, which can then be used to detect cognitive impairment [3–6, 8]. Researchers have also used AI-based analyses to extract disease-specific features from gaze data for diagnosing dementia status [6, 9]. Those metrics are reported to achieve good performance for detecting disease status as a binary outcome (i.e., dementia presence or absence); however, in the clinical setting, obtaining objective and continuous measures of cognitive function (i.e., cognitive scores) that reflect the severity of cognitive decline is crucial for disease monitoring and management. Despite its importance, this type of approach has not yet been fully explored. The ETCA app is designed to provide quantitative and continuous scores reflecting a subject’s cognitive status. These can be used for dementia screening, severity evaluation, and monitoring.

The tablet-based ETCA app presented here adopted task movies designed to assess major cognitive domains similar to the MMSE [5, 22] (Supplementary Fig. 1) and a scoring algorithm that provides continuous scores based on viewing time on the correct image (Fig. 2). This approach can provide scores that are highly correlated with those of the MMSE, thereby enhancing the clinical utility and validity of the ETCA app. Although most previous studies have used research-based eye-tracking devices mounted on a PC monitor, the tablet-based ETCA app uses the built-in camera of a widely used tablet to enable a time-efficient and scalable approach for cognitive assessment. Most importantly, this is the first ETCA app whose performance has been demonstrated in a valid clinical trial with robust data management and an analysis plan.

Significant correlations were observed between the ETCA app and the MMSE subscores, particularly regarding the orientation, attention, and memory tasks (Table 2 and Fig. 4). These cognitive domains are typically impaired in the early stage of dementia and are crucial for early diagnosis [16, 22]. Memory deficit is among the first symptoms reported by patients with dementia, particularly in those with Alzheimer’s disease [23, 24]. The high correlation observed in the memory subscore between the ETCA app and the MMSE suggests that the ETCA app is capable of effectively detecting the earliest signs of dementia. In this trial, we grouped the individual MMSE items into five cognitive domains according to previous research [15–18]. However, psychometric evidence for the MMSE subscores may be insufficient for a proper representation of each cognitive domain. Further validation with specific neuropsychological tests focusing on each cognitive domain will be needed in a future study.

The standard administration time for the MMSE is approximately 10 min [25, 26]. The administration time for neuropsychological tests can be longer for patients with dementia, compared to cognitively normal individuals [27, 28]. Most participants in the present trial completed the ETCA within 4–5 min (Table 3), demonstrating its time-saving advantage for cognitive testing. This reduces the test-related burdens on both participants and administrators. Importantly, similar administration times were observed across subgroups, indicating that the ETCA app can be administered quickly, even to subjects with cognitive impairment.

The ETCA app achieved excellent performance for detecting cognitive impairment and dementia (Fig. 5). The traditional cutoff value of 23/30 for the MMSE has been widely used for detecting patients with dementia [19, 20]. In the present trial, the optimal cutoff values for the ETCA app to detect cognitive impairment (Fig. 5a) and dementia (Fig. 5b) were both 45/100, suggesting that an ETCA app score of 45/100 is equivalent to an MMSE score of 23/30. The ROC analysis revealed a considerable AUC value (0.864) for the ETCA app to detect dementia (Fig. 5b), indicating that the ETCA app is a suitable screening tool.

We noted significant differences in the ETCA app scores among the MMSE subgroups, and the overlap among the subgroups was sufficiently low to discriminate them (Supplementary Fig. 2). This suggests that the ETCA app has an extensive dynamic performance range as a screening tool. The ETCA has a relatively low ceiling effect, owing to its scoring algorithm (i.e., the score is determined as a percentage of the viewing time spent on the correct image) (Fig. 2); thus, obtaining a full score (100%) is impossible, even in individuals with normal cognitive function. This serves to enable the stratification of subjects with relatively high MMSE scores. The low ceiling effect may improve the diagnostic performance for mild cognitive impairment (MCI) detection; this should be explored in future studies.

The study confirmed a significant correlation between the ETCA app and MMSE scores, with only a small number of participants exhibiting divergent scores between the tests (Fig. 3). Several potential factors may account for these score discrepancies. The ETCA primarily relies on visual information, while the MMSE depends on auditory and linguistic inputs. Thus, a divergence in scores might arise in subjects who have discrepancies between their visual and verbal memories. The presence or absence of time constraints for the tests may also cause discrepancies. Subjects with frontal lobe dysfunction who require more time to process thoughts might be able to answer the MMSE correctly, as it has no time limit, but then score lower on the time-constrained ETCA. By contrast, in the interview-style MMSE, subjects who cannot demonstrate their full cognitive abilities due to stress might score lower, but perform better when using the low-stress ETCA. Any interpretation of ETCA results should consider these factors.

This trial has potential limitations. First, it did not consider the clinical diagnostic status of MCI, and it included participants with MCI in the non-demented population. Among the 34 non-demented participants in the FAS population, eight (23.5%) participants had MMSE scores of 27 or lower; therefore, they were suspected to be MCI, based on the cutoff value reported in the previous studies [12, 29]. As MCI is an important target for early intervention to prevent dementia, the ETCA app’s performance for detecting MCI should be explored in future studies. Second, the task movie included only a single task for visuospatial function and language, resulting in relatively low correlation coefficients in those tasks. This may decrease the accuracy of the ETCA app when specifically assessing these cognitive domains. Increasing the number of tasks would, presumably, improve accuracy; however, this would also increase the administration time. Our aim here was to limit the duration of the ETCA’s task movie to three minutes, to enhance its utility as a screening tool. Third, diagnoses of dementia were made mainly based on the examiner’s clinical judgement. Although most participants diagnosed with dementia also underwent detailed structured tests and brain imaging to support the diagnosis, some of the non-demented participants did not. This might have affected the results of the ETCA’s diagnostic performance for dementia. Lastly, we did not evaluate the ETCA’s test reliability, such as its test–retest reliability, in this clinical trial. Test reliability could affect the clinical utility of the ETCA, and these measures should be examined carefully in a future study.

Overall, this clinical trial demonstrated that the tablet-based ETCA app enabled rapid and low-stress cognitive assessment, with scores that were highly correlated with those of the MMSE. The results verified the clinical utility of the ETCA app as a cognitive assessment tool for dementia screening and management. The tablet-based ETCA app has the benefit of being both highly portable and more user-friendly, compared to traditional neuropsychological tests, as a trained examiner is not required for its successful execution. Based on the trial results, Japan’s regulatory authority has officially approved the ETCA app as SaMD for neuropsychological assessment (approval #30500BZX00235000).

## Supplementary Information

Below is the link to the electronic supplementary material.

Supplementary file1 (DOCX 912 KB)

## Data Availability

The datasets used and/or analyzed during the current study are available from the corresponding author upon reasonable request.
