# Supplementary material for: Eye-tracking-based tablet application for enhanced cognitive assessment: a clinical trial for software as a medical device (SaMD)
Source: GeroScience. 2025 Oct 20;48(4):5379–91. doi: 10.1007/s11357-025-01941-x (PMC13575033; doi:10.1007/s11357-025-01941-x)
Supplement: Supplementary file 1 [file 11357_2025_1941_MOESM1_ESM.docx]

**Supplementary information**

**Methods**

**Trial design and participants: registration**

The trial did not impose a specific screening period. Each participant was registered after completing the MMSE assessment. The registration center monitored the MMSE score distribution of the registered participants, to ensure we enrolled an equal number of participants from each MMSE subgroup (see “Sample-size calculation”).

The investigators conducted a cognitive assessment using the MMSE for participants judged eligible to participate. They then informed the registration center of the participant’s MMSE score and registered the participant’s data. Next, they conducted a cognitive assessment using the ETCA app; this was done on the same day the MMSE was administered. Immediately after completing both tests, a questionnaire-based assessment was provided to both the participants and administrators.

**Randomization and masking**

This was a single-arm, open-label, uncontrolled trial without randomization or blind controls.

**Sample-size calculation**

A clinical study of 80 participants (27 healthy controls, 26 MCI, and 27 patients with dementia) conducted at Osaka University Hospital demonstrated a high correlation for the cognitive scores between the ETCA and the MMSE, with a correlation coefficient of 0.74 (*p* < 0.00001, Spearman).^1^ An exploratory clinical study of 69 participants (20 healthy controls, 15 MCI, and 34 patients with dementia) using the ETCA app conducted at Osaka University Hospital demonstrated a high correlation in the total scores between the ETCA app and the MMSE, with a correlation coefficient of 0.813 (*p* < 0.00001, Spearman). Based on these results, and given the expected correlation coefficient of 0.75 with a significance level of 2.5% (one-sided) and 80% power, the investigators assumed that the correlation of the total scores between the ETCA app and the MMSE would have been demonstrated if the lower limit of the two-sided 95% CI of the correlation coefficient was no smaller than 0.55. Based on this calculation, a sample size of 65 was considered sufficient to detect a statistical significance. Assuming a dropout rate of 15%, the required sample size was calculated as 75.

This trial enrolled almost equal numbers of participants in each subgroup, based on MMSE scores of ≤ 19, 20–26, and 27 ≤, thus ensuring the distribution of MMSE scores would be broad and balanced. This enabled us to demonstrate correlations between ETCA app results and MMSE scores in individuals with a wide range of cognitive states, including dementia, MCI, and normal healthy controls. The above-mentioned cutoff scores for the MMSE subgroups were determined based on the results of a previous study^1^ and an exploratory study conducted at Osaka University Hospital.

**Data collection**

Task movies were presented to subjects on a tablet with a screen size of 214.9 × 280.6 mm. The tablet was attached vertically to a stand facing the subject in the portrait orientation, with the front-facing camera at the bottom of the tablet. Image data for eye tracking were collected using the front-facing camera at a sampling rate of 50 Hz. An AI-based eye-tracking method based on the ARKit application programming interfaces (APIs) of the iPadOS was used to estimate the subject’s gaze.

**Data processing**

The subject’s gaze information created by the eye-tracking system was output in comma-separated value (CSV) format (viewpoint: x- and y-coordinates with timeline) and processed into gaze plot data (Fig. 2b, red points represent gaze plot). The percentage of time spent viewing the correct image was calculated based on the number of gaze plots in the ROI (Fig. 2b).

The ETCA app provides an automatic calibration-check system to ensure the accuracy of gaze detection before beginning the cognitive assessment (see “Calibration and calibration-check session”). The calibration check is designed to repeat automatically after each failed case for up to three cycles. Prior clinical research has shown that repeating the calibration for a maximum of 12 times (3 cycles × 4 sets) helps to resolve calibration errors, such as incorrect posture positions. Participants discontinued the study if the ECTA app calibration check was unsuccessful after 12 attempts.

**Preparation for the assessment**

The subject was instructed to sit directly facing the tablet’s screen for the measurement. There was no need to fix the subject’s head in place. The tablet’s position was optimized by positioning the subject’s face (shown on the monitor and captured with the front-facing camera) inside an elliptical frame that appeared on the screen as a guide. The tablet was placed approximately 51 cm away from the subject’s eyes. All assessments were performed in a standard clinical examination room under natural lighting.

Before beginning the test, the subject was given the following instructions: 1) The test involves only watching task videos; you do not need to respond verbally or point to answers. 2) There will first be a calibration session, followed by task videos totaling about three minutes. During the calibration procedure, you will look at images displayed on the screen. 3) Following the instructions provided during the task videos, locate the correct answer and look at it. 4) Try to minimize your head and body movements during the test. The task videos had accompanying background music, but no audio instructions were provided.

**Calibration and calibration-check session**

A calibration video was shown first to match the participant’s gaze point, as captured by eye tracking, with coordinates on the tablet (Supplementary Fig. 1a). A circular target was shown in the center of the screen, with text instructing the subject to look at it. Next, the circular target moved to the upper left of the screen, where it flashed for three seconds. Similarly, it moved to the upper right and flashed for three seconds, then to the lower right and flashed for three seconds, and finally to the lower left for three seconds.

After calibration, a calibration check video was shown to check eye-tracking accuracy (Supplementary Fig. 1b). First, a picture of an apple was shown in the upper right corner of the screen, along with the text “Focus on the apple at the top right.” The instructions disappeared and the subject looked at the apple in the upper right corner for five seconds. In the same way, the apple was shown in the bottom left corner of the screen with the text “Focus on the apple at the bottom left.” Again, the instructions disappeared and the subject looked at the apple for five seconds. When the X–Y gaze coordinates, as determined through eye tracking, were judged close enough to the target’s position on the screen (i.e., the center of the apple), the task videos for cognitive function evaluation were initiated. Specifically, eye tracking was deemed sufficiently accurate when the averaged gaze point coordinates, while looking at the apple, were within 200 pixels of the coordinates of the apple’s center. This calibration process was repeated if eye tracking was deemed insufficiently accurate.

**Task movies and pictures for the assessment of cognitive function**

The task movies were designed to evaluate the cognitive function domains of attention/judgment, visuospatial function, language, memory (delayed recall), and orientation (place and time) (Supplementary Fig. 1). Each task video showed instruction or problem text and four choices that appeared simultaneously. One of the four choices was the correct answer. An ROI was set for the correct answer, and the percentage of the subject’s viewing time spent in the ROI was recorded as their score. The task movies were shown on the tablet in the following order (running for about three minutes):

**Memory (encoding)**

Four pictures were shown for 10 seconds each, along with text instructing the subject to remember each of them (Supplementary Fig. 1c).

**Attention and judgment**

Pictures representing four choices were shown for seven seconds, with text instructing the subject to “Focus on the odd one out.” One of the four pictures was conceptually different from the others, making it the correct choice (Supplementary Fig. 1d). After seven seconds for evaluation, the correct choice was shown in a red frame for two seconds.

Next, a calculation problem (subtracting 5 from 94) was shown on the screen for five seconds (Supplementary Fig. 1e). The calculation problem text then disappeared and four choices, one of which was correct, were shown for five seconds. The correct choice was then shown in a red frame for two seconds.

A second calculation problem (subtracting 5 from 71) was then shown for five seconds (Supplementary Fig. 1f). Three seconds after the problem text disappeared, four choices, one of which was correct, were displayed for five seconds. For three seconds after the question disappeared, until the choices appeared, the subject needed to keep the answer in mind. The correct choice was finally shown in a red frame for two seconds.

**Visuospatial function**

First, a shape was shown on the screen for six seconds with the instruction, “Remember this shape.” The screen then changed to show the text “Focus on the shape you just saw” with four shape choices, including the correct one, for six seconds (Supplementary Fig. 1g). The correct choice was finally shown in a red frame for two seconds.

**Language (naming)**

The subject was first shown the problem text “What is this called?” and an item (a pencil) for five seconds, along with four words, one of which was the correct answer (Supplementary Fig. 1h). The problem text disappeared, leaving only the item and the four choices displayed for five seconds. The correct choice was finally shown in a red frame for two seconds.

**Memory (delayed recall)**

This session evaluated the degree to which the picture memorized earlier for “Memory (encoding)” was remembered. First, the instruction text “Focus on the picture you remembered earlier” was shown for seven seconds, along with four choices, one of which was the correct answer (Supplementary Fig. 1i). The correct choice was finally shown in a red frame for two seconds.

**Orientation (place)**

The instruction text “Focus on the place where you are now” was shown for eight seconds, along with four choices representing locations, one of which was the correct answer (Supplementary Fig. 1j). The correct choice was finally shown in a red frame for two seconds.

**Orientation (time)**

The instruction text “Focus on the current month” was shown for eight seconds, along with four choices representing months, one of which was the correct answer (Supplementary Fig. 1k). The correct choice was finally shown in a red frame for two seconds.

**Questionnaire-based assessments for participants and administrators**

The questionnaire-based assessments for administrators regarding the ease of use of the ETCA app were developed in two phases: the first was a simple questionnaire administered after each assessment, the second was a detailed reflective questionnaire after the completion of the entire study.

The participants’ questionnaire asked about fatigue during the ETCA app assessment, compared with the MMSE (options: “felt less fatigued than during the MMSE,” “felt the same as during the MMSE,” and “felt more fatigued than during the MMSE”) and mental stress during the ETCA, compared with the MMSE (options: “felt less mental stress compared with the MMSE,” “felt the same mental stress as with the MMSE,” and “felt more mental stress than with the MMSE”).

The administrators’ questionnaire asked about ease of using the ETCA app, compared with the MMSE (options: “easier,” “a little bit easier,” “same,” “less easy,” and “much less easy”). The detailed reflective questionnaire asked about the ease of using the ETCA app, compared to the MMSE (options: “easier,” “a little bit easier,” “same,” “less easy,” and “much less easy”); ease of activating/completing the ETCA app (options: “easier,” “a little bit easier,” “same,” “less easy,” and “much less easy”); and their views on the ETCA as an alternative to the MMSE (options: “could be used as an alternative to the MMSE,” “hard to say,” and “cannot be used as an alternative to the MMSE”).

**Reference**

1	Oyama, A. *et al.* Novel Method for Rapid Assessment of Cognitive Impairment Using High-Performance Eye-Tracking Technology. *Scientific Reports* 9, 12932, doi:10.1038/s41598-019-49275-x (2019).

**Supplementary Figure 1. Task movies**

See the supplementary methods for details.

**Supplementary Figure 2. ETCA scores in each MMSE subgroup**

The boxplots show the median, interquartile range, and extreme values of the ETCA app scores in each MMSE subgroup: ≤ 19 (n = 28), 20–26 (n = 21), and 27 ≤ (n = 24); *****p* < 0.0001, the Kruskal–Wallis test followed by the Steel–Dwass multiple comparison test.

**Supplementary Table 1. Detailed demographics and baseline characteristics (FAS population)**

|  |  | MMSE ≤ 19 | MMSE 20–26 | 27 ≤ MMSE | All |
| --- | --- | --- | --- | --- | --- |
|  | Statistics | (N = 28) | (N = 21) | (N = 24) | (N = 73) |
|  |  |  |  |  |  |
| **Age** | n | 28 | 21 | 24 | 73 |
|  | Mean | 78.5 | 79.7 | 74.0 | 77.4 |
|  | SD | 5.7 | 4.0 | 10.8 | 7.7 |
|  | Min | 64 | 71 | 48 | 48 |
|  | Median | 80.0 | 80.0 | 77.5 | 79.0 |
|  | Max | 86 | 87 | 89 | 89 |
|  |  |  |  |  |  |
| **Age category** |  |  |  |  |  |
| 20–29 | n (%) | 0 (0.0%) | 0 (0.0%) | 0 (0.0%) | 0 (0.0%) |
| 30–39 | n (%) | 0 (0.0%) | 0 (0.0%) | 0 (0.0%) | 0 (0.0%) |
| 40–49 | n (%) | 0 (0.0%) | 0 (0.0%) | 1 (4.2%) | 1 (1.4%) |
| 50–59 | n (%) | 0 (0.0%) | 0 (0.0%) | 2 (8.3%) | 2 (2.7%) |
| 60–69 | n (%) | 1 (3.6%) | 0 (0.0%) | 3 (12.5%) | 4 (5.5%) |
| 70–79 | n (%) | 11 (39.3%) | 10 (47.6%) | 10 (41.7%) | 31 (42.5%) |
| 80–89 | n (%) | 16 (57.1%) | 11 (52.4%) | 8 (33.3%) | 35 (47.9%) |
| ≥90 | n (%) | 0 (0.0%) | 0 (0.0%) | 0 (0.0%) | 0 (0.0%) |
|  |  |  |  |  |  |
| **Age category** |  |  |  |  |  |
| ≤64 | n (%) | 1 (3.6%) | 0 (0.0%) | 5 (20.8%) | 6 (8.2%) |
| ≥65 | n (%) | 27 (96.4%) | 21 (100.0%) | 19 (79.2%) | 67 (91.8%) |
|  |  |  |  |  |  |
| **Sex** |  |  |  |  |  |
| Male | n (%) | 7 (25.0%) | 8 (38.1%) | 15 (62.5%) | 30 (41.1%) |
| Female | n (%) | 21 (75.0%) | 13 (61.9%) | 9 (37.5%) | 43 (58.9%) |
|  |  |  |  |  |  |
| **Education history (years)** | n | 28 | 21 | 24 | 73 |
|  | Mean | 11.9 | 11.8 | 14.0 | 12.6 |
|  | SD | 2.3 | 2.1 | 3.1 | 2.7 |
|  | Min | 9 | 9 | 9 | 9 |
|  | Median | 12.0 | 12.0 | 14.0 | 12.0 |
|  | Max | 16 | 16 | 21 | 21 |
|  |  |  |  |  |  |
| **Education history category (years)** |  |  |  |  |  |
| ≤15 | n (%) | 24 (85.7%) | 19 (90.5%) | 14 (58.3%) | 57 (78.1%) |
| ≥16 | n (%) | 4 (14.3%) | 2 (9.5%) | 10 (41.7%) | 16 (21.9%) |
|  |  |  |  |  |  |
| ≤9 | n (%) | 8 (28.6%) | 5 (23.8%) | 2 (8.3%) | 15 (20.5%) |
| 10–15 | n (%) | 16 (57.1%) | 14 (66.7%) | 12 (50.0%) | 42 (57.5%) |
|  |  |  |  |  |  |
| **Dementia status** |  |  |  |  |  |
| Dementia | n (%) | 25 (89.3%) | 14 (66.7%) | 0 (0.0%) | 39 (53.4%) |
| Not dementia | n (%) | 3 (10.7%) | 7 (33.3%) | 24 (100.0%) | 34 (46.6%) |
|  |  |  |  |  |  |
| **Time of diagnosis (years ago)** |  |  |  |  |  |
| n = number with dementia | n | 25 | 14 | 0 | 39 |
|  | Mean | 3.2 | 1.7 | - | 2.7 |
|  | SD | 1.8 | 1.2 | - | 1.7 |
|  | Min | 0 | 0 | - | 0 |
|  | Median | 3.0 | 1.5 | - | 3.0 |
|  | Max | 7 | 4 | - | 7 |

**Supplementary Table 2. Questionnaire-based assessments of the ease of use of the ETCA app (FAS population)**

|  |  | All |
| --- | --- | --- |
|  | Statistics | (N = 73) |
|  |  |  |
| The ETCA app's ease of use, compared to MMSE |  |  |
| Easier | n (%) | 62 (84.9%) |
| A little bit easier | n (%) | 9 (12.3%) |
| Same | n (%) | 2 (2.7%) |
| Less easy | n (%) | 0 (0.0%) |
| Much less easy | n (%) | 0 (0.0%) |

**Supplementary Table 3. Questionnaire-based assessments for administrators regarding the usability of the ETCA app**

|  |  | All |
| --- | --- | --- |
|  | Statistics | (N = 4) |
|  |  |  |
| Ease of using the ETCA app, compared with the MMSE | | |
| Easier | n (%) | 4 (100.0%) |
| A little bit easier | n (%) | 0 (0.0%) |
| Same | n (%) | 0 (0.0%) |
| Less easy | n (%) | 0 (0.0%) |
| Much less easy | n (%) | 0 (0.0%) |
|  |  |  |
| Ease of activating/completing the ETCA app | |  |
| Easy | n (%) | 3 (75.0%) |
| A little bit easy | n (%) | 1 (25.0%) |
| Hard to say | n (%) | 0 (0.0%) |
| A little bit difficult | n (%) | 0 (0.0%) |
| Difficult | n (%) | 0 (0.0%) |
|  |  |  |
| Possible alternative to MMSE |  |  |
| Could be used as an alternative to the MMSE | n (%) | 4 (100.0%) |
| Hard to say | n (%) | 0 (0.0%) |
| Coud not be used as an alternative to the MMSE | n (%) | 0 (0.0%) |

N = number of administrators

**Supplementary Table 4. Adverse events (SAF population)**

|  | N = 78 (SAF population) | | | | | | | | | | | |
| --- | --- | --- | --- | --- | --- | --- | --- | --- | --- | --- | --- | --- |
|  | Before the test | | | During the test | | | After the test | | | All | | |
|  | n | (%) | count | n | (%) | count | n | (%) | count | n | (%) | count |
|  |  |  |  |  |  |  |  |  |  |  |  |  |
| Number of participants with |  |  |  |  |  |  |  |  |  |  |  |  |
| Any adverse event to the participant related to the  ETCA app assessment | 0 | (0.0) | 0 | 1 | (1.3) | 1 | 0 | (0.0) | 0 | 1 | (1.3) | 1 |
| Any adverse event to the participant | 0 | (0.0) | 0 | 1 | (1.3) | 1 | 0 | (0.0) | 0 | 1 | (1.3) | 1 |
| Any serious adverse event to the participant | 0 | (0.0) | 0 | 0 | (0.0) | 0 | 0 | (0.0) | 0 | 0 | (0.0) | 0 |
| Any high-severity adverse event to the participant | 0 | (0.0) | 0 | 0 | (0.0) | 0 | 0 | (0.0) | 0 | 0 | (0.0) | 0 |
|  |  |  |  |  |  |  |  |  |  |  |  |  |
| Any adverse event to someone other than the participant | 0 | (0.0) | 0 | 0 | (0.0) | 0 | 0 | (0.0) | 0 | 0 | (0.0) | 0 |

Mild neck pain was observed in one participant during the test, but the participant recovered within the day without any treatment.

**Supplementary Table 5. Malfunctions of the ETCA app (SAF population)**

|  | N = 78 (SAF population) | | | | | | | | | | | |
| --- | --- | --- | --- | --- | --- | --- | --- | --- | --- | --- | --- | --- |
|  | Before the test | | | During the test | | | After the test | | | All | | |
|  | n | (%) | count | n | (%) | count | n | (%) | count | n | (%) | count |
|  |  |  |  |  |  |  |  |  |  |  |  |  |
| Number of participants with |  |  |  |  |  |  |  |  |  |  |  |  |
| Any adverse event to the participant related to the ETCA app assessment | 0 | (0.0) | 0 | 1 | (1.3) | 1 | 0 | (0.0) | 0 | 1 | (1.3) | 1 |
| Any adverse event to the participant | 0 | (0.0) | 0 | 1 | (1.3) | 1 | 0 | (0.0) | 0 | 1 | (1.3) | 1 |
| Any serious adverse event to the participant | 0 | (0.0) | 0 | 0 | (0.0) | 0 | 0 | (0.0) | 0 | 0 | (0.0) | 0 |
| Any high-severity adverse event to the participant | 0 | (0.0) | 0 | 0 | (0.0) | 0 | 0 | (0.0) | 0 | 0 | (0.0) | 0 |
|  |  |  |  |  |  |  |  |  |  |  |  |  |
| Any adverse event to someone other than the participant | 0 | (0.0) | 0 | 0 | (0.0) | 0 | 0 | (0.0) | 0 | 0 | (0.0) | 0 |
|  |  |  |  |  |  |  |  |  |  |  |  |  |
